# Short term increase in low birth weight babies after Fukushima

**DOI:** 10.1186/s12940-020-00650-6

**Published:** 2020-11-25

**Authors:** Alfred Körblein

**Affiliations:** Nürnberg, Germany

**Keywords:** Fukushima, Low birth weight, Logistic regression, Trend analysis

## Abstract

An analysis of low birth weight (LBW) births in ten contaminated prefectures of Japan, 1995–2018, finds a statistically significant increase in the LBW proportion in 2012–2013, but no increase after 2013. In the rest of Japan (37 prefectures), no increase in LBW births was found after the Fukushima accident.

In their recent article, Scherb et al. [1] investigated annual data of live births with low birth weight (LBW), 1995–2018, in selected regions of Japan before and after the Fukushima nuclear accident in March 2011. The trend of LBW-proportion was analyzed employing logistic regression with a fourth-degree polynomial for the temporal trend, and allowing for a level shift from 2012 onward (variable cp, defined as cp = 1 in 2012–2018 and cp = 0 otherwise). Results were presented for Japan as a whole and three sub-areas: (1) five highly contaminated prefectures (Fukushima, Miyagi, Ibaraki, Tochigi, and Iwate), (2) five moderately contaminated prefectures (Yamagata, Saitama, Tokyo, Kanagawa, and Chiba), and (3) the rest of Japan (37 prefectures). In Japan as a whole, the level shift was statistically significant (OR = 1.020, *p*-value 0.025). The effect was greater in area 1 (OR = 1.055, *p*-value 0.010) than in area 2 (OR = 0.021, *p*-value 0.011), and not statistically significant in area 3 (OR = 1.015, *p*-value 0.105).

Since the numbers of live births and LBW births for all three areas were provided in Table 1 of [1], I was able to check the results. In my analysis I used logistic regression with program R (https://www.r-project.org/); the option family = quasibinomial was applied to adjust the variances for overdispersion. This means that F-tests were used instead of chi-square tests.

With a fourth-degree polynomial for the time trend as applied in [1], the estimate for the odds ratio (OR) of the level shift in the data from Japan as a whole was OR = 1.018, *p*-value 0.074. A fifth-degree polynomial for the temporal trend, however, fitted the data better (deviance = 74.1, df = 17) than a fourth-degree polynomial (deviance = 90.8, df = 18). Now, the odds ratio for the level shift decreased to OR = 1.009 (95% CI: 0.991, 1.028), *p*-value 0.347. Similarly, the regressions of the data for the three sub-areas yielded non-significant level shifts when a fifth-degree polynomial was applied: area 1: OR = 1.039 (0.988, 1.092), *p*-value 0.152; area 2: OR = 1.013 (0.992, 1.035), *p*-value 0.244; area 3: OR = 1.004 (0.982, 1.026), *p*-value 0.730). For all three data sets, a fifth-degree polynomial yielded a better fit than a fourth-degree polynomial.

To increase the statistical power, I pooled the data from areas 1 and 2 and termed them the study region; area 3 was used as the control region. The odds ratio of the level shift in the study region was 1.019 (0.994, 1.044), *p*-value 0.152. No notable level shift was detected in the control region (OR = 1.004, *p*-value 0.73).

A plot of the data shows an increase in LBW-proportions in 2012–2013. Therefore an additional regression analysis was carried out with a model that allowed for a short term increase in 2012–2013 (model 2) instead of a level shift in 2012–2018 (model 1). The estimate of the odds ratio in 2012–2013 was 1.016 (1.002, 1.031), *p*-value 0.041). The deviance was 40.2 (df = 17), considerably smaller than deviance = 45.9 (df = 17) obtained with model 1. A similar regression for the control region with model 2 yielded OR = 0.998 (*p*-value 0.76). The trend of LBW proportions in the study region and the regression lines for the two competing models are shown in Fig. 1, upper and lower panel.

**Fig. 1:**
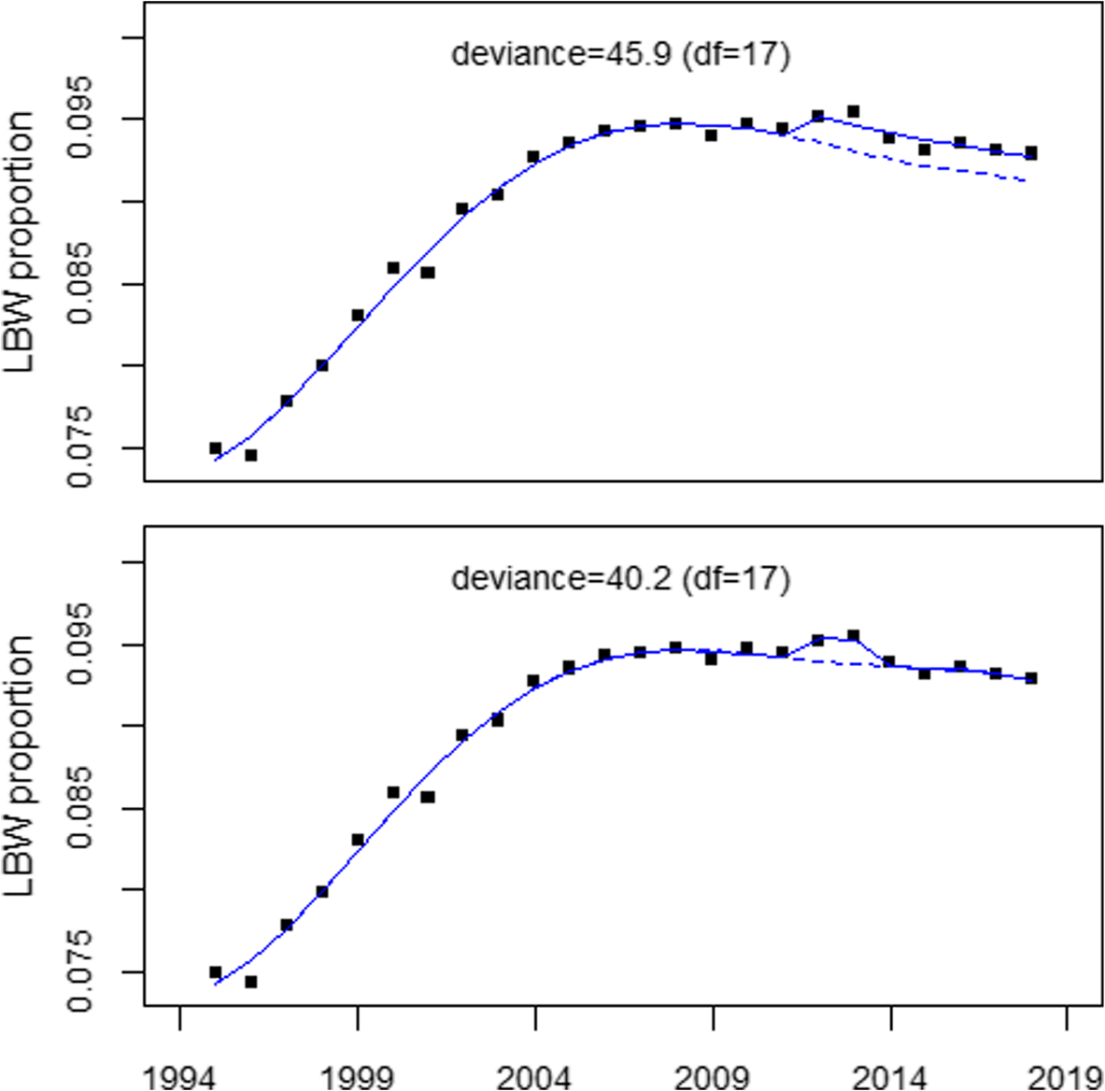
Low birth weight proportions in the study region, 1995–2018, and results of regressions allowing for a level shift in 2012 (upper panel) and for an increase in 2012–2013 (lower panel)

A considerable improvement of the fit was obtained when 2002 was chosen as the beginning of the study period. Logistic regression with a fourth-degree polynomial turned out to be the best choice for the long-term trend. The results were statistically significant for both regression model 1 (OR = 1.019 (1.003, 1.035), *p*-value 0.018) and model 2 (OR = 1.015 (1.005, 1.025), *p*-value 0.002), but model 2 fitted the data better (deviance = 7.5, df = 11) than model 1 (deviance = 11.3, df = 11). As the data showed no overdispersion, a chi-square test was applied for testing the significance of the odds ratios. The test for a possible level shift in addition to the effect in 2012–2013 yielded OR = 1.00 (*p*-value 0.93). Thus, the significant result for the shift in LBW proportion obtained with model 1 is driven by the peak in 2012–2013.

To conclude, my re-analysis of the data shows that there was an increase in LBW proportion in the first 2 years after Fukushima, but no increase after 2013. Thus, the claim by Scherb et al. that their result is evidence of a genetic radiation effect is challenged by the present analysis.

## Data Availability

Data was made available in reference [1].

## References

[1] Scherb H, Hayashi K (2020). Spatiotemporal association of low birth weight with Cs-137 deposition at the prefecture-level in Japan after the Fukushima nuclear power plant accidents: an analytical-ecologic epidemiological study. Environ Health.

